# Can transplant renal scintigraphy predict the duration of delayed graft function? A dual center retrospective study

**DOI:** 10.1371/journal.pone.0193791

**Published:** 2018-03-21

**Authors:** Stan Benjamens, Robert A. Pol, Lioe-Fee de Geus-Oei, Aiko P. J. de Vries, Andor W. J. M. Glaudemans, Stefan P. Berger, Riemer H. J. A. Slart

**Affiliations:** 1 Department of Surgery, Division of Transplant Surgery, University of Groningen, University Medical Center Groningen, Groningen, The Netherlands; 2 Medical Imaging Center, University of Groningen, University Medical Center Groningen, Groningen, The Netherlands; 3 Department of Radiology, Division of Nuclear Medicine, Leiden University, Leiden University Medical Center, Leiden, The Netherlands; 4 Department of Internal Medicine, Division of Nephrology, and Leiden Transplant Center, Leiden University, Leiden University Medical Center, Leiden, The Netherlands; 5 Department of Internal Medicine, Division of Nephrology, University of Groningen, University Medical Center Groningen, Groningen, The Netherlands; 6 Department of Biomedical Photonic Imaging, University of Twente, Enschede, The Netherlands; Universita degli Studi di Perugia, ITALY

## Abstract

**Introduction:**

This study focused on the value of quantitatively analyzed and qualitatively graded renal scintigraphy in relation to the expected duration of delayed graft function after kidney transplantation. A more reliable prediction of delayed graft function duration may result in a more tailored and patient-specific treatment regimen post-transplantation.

**Methods:**

From 2000 to 2014, patients with early transplant dysfunction and a Tc-99m MAG3 renal scintigraphy, within 3 days post-transplantation, were included in a dual center retrospective study. Time-activity curves of renal scintigraphy procedures were qualitatively graded and various quantitative indices (*R20/3*, *TFS*, *cTER*, *MUC10*) were combined with a new index (*Average upslope)*. The delayed graft function duration was defined as the number of days of dialysis-based/functional delayed graft function.

**Results:**

A total of 377 patients were included, with a mean age (± SD) of 52 ± 14 years, and 58% were male. A total of 274 (73%) patients experienced delayed graft function≥ 7 days. Qualitative grading for the prediction of delayed graft function≥ 7 days had a sensitivity and specificity of respectively 87% and 65%. The quantitative indices with the most optimal results were *cTER* (76% sensitivity, 72% specificity), and *Average upslope* (75% sensitivity, 73% specificity).

**Conclusions:**

Qualitative renal scintigraphy grading and the quantitative indices *cTER* and *Average upslope* predict delayed graft function ≥ 7 days with a high sensitivity. This finding may help to support both clinicians and patients in managing early post-operative expectations. However, the specificity is limited and thus renal scintigraphy does not reliably help to identify patients in whom the course of delayed graft function is longer than anticipated.

## Introduction

Monitoring renal function after kidney transplantation (KTX) is pivotal to recognize post-transplant complications, such as vascular or urological complications, acute tubular necrosis (ATN) or rejection.[1] Therefore, an imaging modality with a high predictive value for post-KTX complications may result in a more tailored and patient-specific treatment regimen and possible reduction of early graft failure.

Delayed graft function (DGF) is a common complication after KTX, with an overall incidence between 2% to 50%, depending on the type of kidney graft (donation after circulatory death (DCD), donation after brain death (DBD) or after living donation) and the definition used for DGF.[2] The most commonly used definitions for DGF are (1) dialysis-based DGF, the need for postoperative dialysis within the first 7 days after KTX and (2) functional DGF, defined by a serum creatinine level failing to decrease with 10% on 3 consecutive days following KTX.[3,4] The impact of DGF on the short-term outcome after KTX is a longer duration of hospitalization, increased risk of graft loss and a higher mortality.[5–7]

Renal scintigraphy (RS) is a common and widely used test to assess graft function and complications after KTX. Previous studies introduced RS as a tool for the evaluation and/or prediction of DGF.[8–11] However, correct acquisition and interpretation is difficult, since several different radiopharmaceuticals and multiple quantitative indices are available.[12] In this study we focus on Technetium-99m mercaptoacetyltriglycine (MAG3) RS, the most commonly used radiopharmaceutical for post-KTX evaluation.[13] We evaluate the qualitative grading scale of Heaf and Iversen and the following four quantitative indices: the ratio of uptake at 20 and 3 minutes (R20/3), the tubular function slope (TFS; counts/second), the corrected tubular extraction rate (cTER; mL/min/1.73 m2) and the uptake within the first 10 minutes, as a fraction of the injected dose (MUC10; counts/second/MBq), which showed to correlate significantly with post-KTX outcomes, in studies with smaller cohorts. [8,10,14,15]

Earlier studies showed that RS cannot differentiate reliably between rejection and acute tubulus necrosis (ATN), as a cause of DGF. [16,17] We postulated that RS is able to predict the expected time course of recovery from DGF and may provide useful information to identify patients with additional allograft pathology. Improving the predictive value and clinical applicability of qualitative and quantitative RS indices could result in improved identification of the cause and expected duration of early graft dysfunction. This may facilitate to take image guided treatment decisions, subsequently leading to a reduction in the number of diagnostic biopsies and faster treatment.

We performed a dual center retrospective study to evaluate the prognostic performance of RS, with regard to the duration of DGF after KTX, by using qualitative and quantitative analysis methodology.

## Patients and methods

Patients were included in the University Medical Center Groningen (UMCG) from 2000 to 2014 (*n* = 177) and in Leiden University Medical Center (LUMC) from 2011 to 2014 (*n* = 200). All available post-KTX RS procedures performed within 3 days after KTX were included and patients’ charts were screened for baseline characteristics and renal function within the first year, using the electronic hospital registries. At the UMCG cohort, RS was performed when transplant dysfunction was clinically suspected. In the LUMC cohort, RS was performed according to the standard post-KTX protocol, including cases with suspicion of vascular or urological complications and with ongoing DGF. Primary outcome was the duration of DGF, analyzed as a continuous and dichotomous variable. For dichotomous analyses, patients of both centers were divided into two groups based on graft function: (I) DGF, dialysis-based delayed graft function or functional delayed graft function for ≥ 7 days (*n* = 274); (II) non-DGF, no dialysis-based delayed graft function or functional delayed graft function for ≥ 7 days (*n* = 103). In both centers kidney biopsies were performed per protocol if DGF persisted for more than 7–10 days without sign of improvement. As acute rejection is generally not thought to subside spontaneously it seems improbable that a significant number of rejections were missed with this approach. Patient data were processed and electronically stored according to the declaration of Helsinki Ethical principles for medical research involving human subjects. The local Medical Ethical Committees of both participating medical centers gave approval for this study and waived the need for written informed consent (Medical Ethical Committee, University Medical Center Groningen 2015/338 and Medical Ethical Committee, Leiden University Medical Center P15.372). The clinical and research activities are consistent with the Principles of the Declaration of Istanbul as outlined in the 'Declaration of Istanbul on Organ Trafficking and Transplant Tourism’.

All Tc-99m MAG3 RS procedures were performed using an intravenous administration dose in a range of 80–100 MBq (2.16–2.70 mCi) and results were reanalyzed for the purpose of this study. Dynamic images were acquired with 1-second frames for two minutes (perfusion phase) followed by 20 seconds frames for 28 minutes (clearance phase). Reconstructed RS data were processed using Syngo.via (Siemens Medical Systems, TN, U.S.A.) and regions of interest (ROIs) were drawn manually. Correction for patients’ motions was applied when needed. The renal ROI was drawn proximal to the renal cortex and the background ROI was drawn as a crescent shape, opposite of the renal artery and vein, resulting in a background corrected time-activity curve, from start of the procedure up to 20 minutes. Patients were excluded if RS procedures could not be reanalyzed since re-analysis of the original data, to derive all quantitative indices, was necessary for this study.

**Fig 1:**
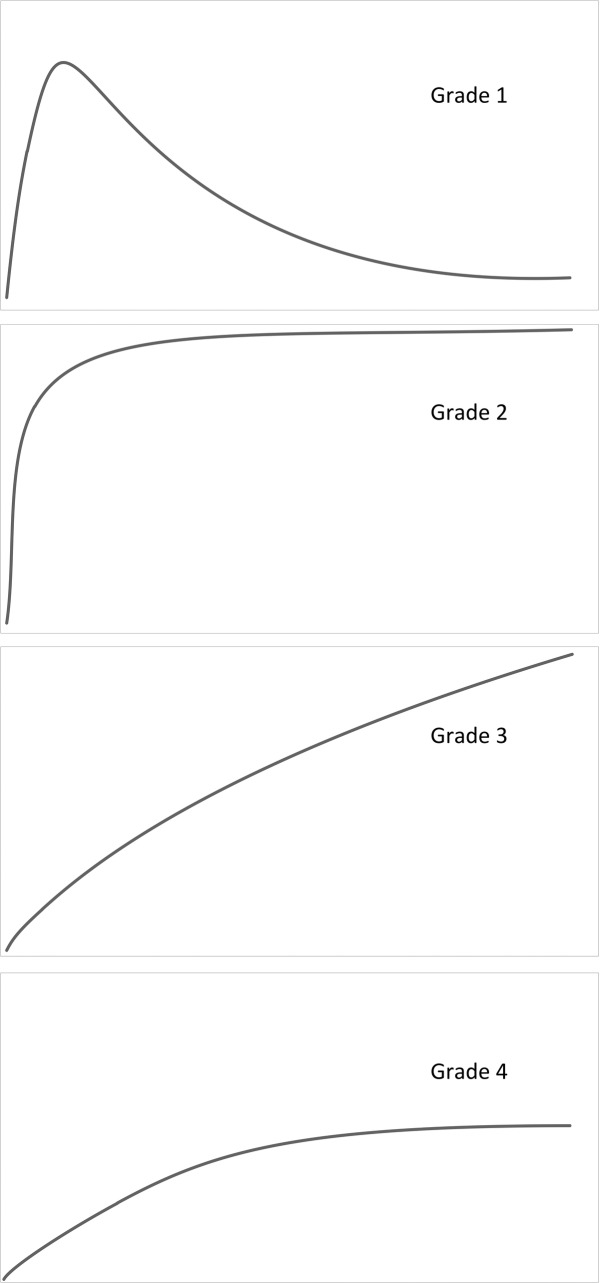
Qualitative renal scintigraphy grading scale for post-KTX Tc-99m MAG3 renal scintigraphy. *Grade 1*, a normal renal scintigraphy curve, with fast uptake and excretion. *Grade 2*, normal uptake with flat excretion curve / plateau phase. *Grade 3*, rising curve without excretion phase. *Grade 4*, reduced absolute uptake without excretion phase.

Time-activity curves were qualitatively graded using the adjusted qualitative grading scale of Heaf and Iversen (Fig 1). This qualitative grading scale divides RS curves based on uptake and excretion curve shapes.[14] For this study we combined the first two and final two grades of the qualitative grading scale of Heaf and Iversen, since the difference between these grades was small with an equal distribution of DGF and non-DGF patients. This resulted in the following grades: *Grade 1*, a normal RS curve, with fast uptake and excretion; *Grade 2*, normal uptake with flat excretion curve; *Grade 3*, rising curve without excretion phase; *Grade 4*, reduced absolute uptake without excretion phase.

**Fig 2:**
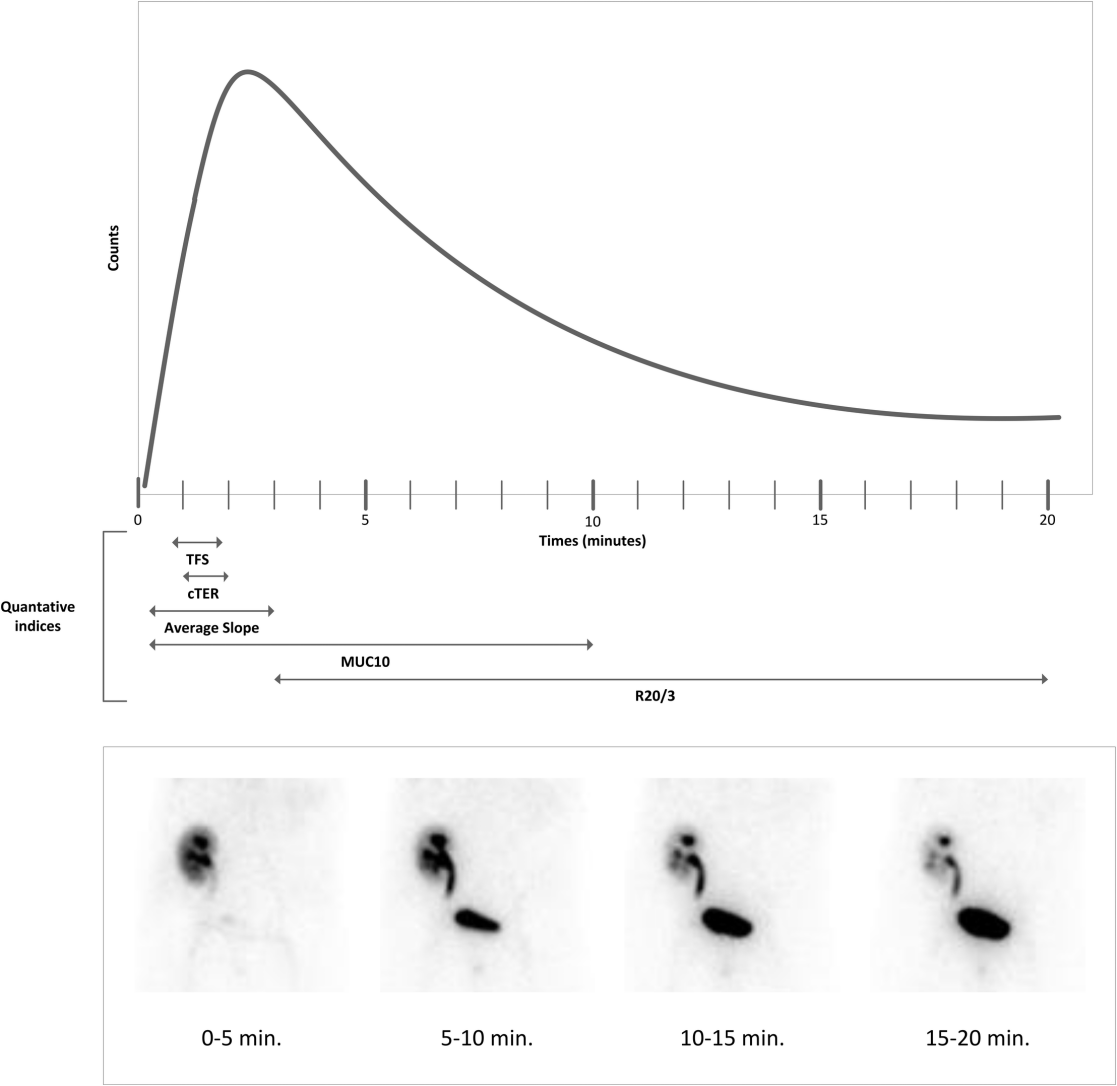
Quantitative indices for post-KTX renal scintigraphy shown for a normal curve, with the corresponding planar RS images. Tubular Function Slope (*TFS*), the linear fit of the curve between 50 and 110 seconds. Corrected tubular extraction rate *(cTER)*, the renal uptake rate for 1 to 2 minutes corrected for body surface. *Average upslope*, the slope between counts at 20 seconds and counts at 3 minutes. *MUC10* resembles the uptake within 10 minutes as a fraction of the injected dose. *R20/3*, retention to uptake ratio, dividing uptake at 20 minutes by the uptake at 3 minutes.

Quantitative assessment of RS curves was performed with the four most common indices *R20/3*, *TFS*, *cTER*, *MUC10*, including a newly developed index *Average upslope* (Fig 2). The *R20/3* index is a quantitative relationship of retention to uptake ratio, which can be calculated by dividing the Tc-99m MAG3 uptake at 20 minutes by the uptake at 3 minutes. [18,19,20] The tubular function slope (*TFS*) reflects the Tc-99m MAG3 uptake by renal tubular cells during the first minutes after Tc-99m MAG3 injection, presented as the linear fit of the curve between 50 and 110 seconds. [8,11] The corrected tubular extraction rate (cTER), in mL/min/1.73m^2^, reflects the Tc-99m MAG3 uptake corrected for the body surface using the renal uptake rate for 1 to 2 minutes after injection and the following regression equation: 9.825X + 11.258 (wherein X is the renal uptake).[15] *MUC10* is an index describing the Tc-99m MAG3 uptake, within the first 10 minutes, as a fraction of the injected dose.[10] Since we hypothesized that the upslope of the RS curve is most influenced by the early graft function, we introduced a new quantitative index, *Average upslope*, reflecting fast rise of the RS curve. The upslope of the RS curve ended between 2 and 3 minutes in the current cohort of Tc-99m MAG3 procedures and this resulted in the following formula: (counts at 3 minutes—counts at 20 seconds) / 160 seconds).

### Statistical analysis

Baseline characteristics and clinical follow-up results are presented as mean and standard deviation (SD) when normal distribution was assumed by means of a Q-Q plot or histogram, as median and interquartile range (IQR) for skewed data and as frequency and percentage when data were categorical. We compared baseline characteristics of both centers, by performing student-t test or chi-square test. We investigated the correlation of qualitative grades and quantitative indices and duration of DGF, by performing a Spearman coefficient of correlation Test. To assess the predictive value of the adjusted qualitative grading scale of Heaf and Iversen, we calculated the number of patients with DGF for each grade and the sensitivity / specificity, and positive / negative predictive value. To assess differences in distribution of the quantitative indices between the DGF patients and the non-DGF patients we performed a Mann-Whitney U test for each index (two-sided p-value < 0.05 = significant), since the quantitative indices were not normally distributed. A receiver-operating characteristic (ROC) curve analysis was used for each significant quantitative parameter, considering an AUC between 0.5–0.7 as a poor level of discrimination, between 0.7–0.8 as an acceptable level of discrimination, and above 0.8 as a reliable level of discrimination.[21] The highest value of the Youden-Index (YI) (sensitivity + specificity-1) was considered as the optimal cut-off point (YI of 1 represents a perfect diagnostic test and YI of 0 indicates that the diagnostic test is not effective).[22] The Kaplan–Meier method was used to visualize the differences between patients with RS-values below or above the optimal cut-off. Differences between Kaplan-Meier curves were determined with the use of log-rank tests. Final results are presented as sensitivity / specificity, and positive / negative predictive value. All statistical analyses were performed with the Statistical Package for the Social Sciences (IBM SPSS Statistics Version 22).

## Results

### Baseline characteristics

In the UMCG, a total of 1690 kidney transplantations were performed from 2000 to 2014 and in the LUMC a total of 511 kidney transplantations were performed from 2011 to 2014. A total of 232 (13.7%) out of the 1690 patients in the UMCG and 200 (39.1%) out of 511 patients in the LUMC fitted the inclusion criteria. Subsequently, 377 patients were included after exclusion of 55 patients due to missing original RS data. Mean age (± SD) was 49 ± 14 and 55 ± 13 years, 57% and 59% were male, 17 (10%) and 24 (12%) patients underwent a preemptive KTX, and the median (IQR) time of (hemo)dialysis prior to transplantation was 32.0 (48.50–54.5) and 36.4 (14.3–57.3) months, respectively, for the UMCG cohort and LUMC cohort (Table 1). In the UMCG cohort, 17% of patients were transplanted after living donation, 41% after donation after brain death (DBD), and 42% after donation after circulatory death (DCD). In the LUMC cohort, living donation was seen in 16%, DBD in 25%, DCD in 47%, and simultaneous kidney-pancreas donation in 12% of patients. A total of 143 (81%) and 131 (66%) patients developed DGF for a duration of > 7 days respectively, in the UMCG cohort and LUMC cohort. Hundred-twenty-six patients (46.0%) were stratified in the DGF > 7 days group based on dDGF and 148 (54.0%) patients were stratified in this group based on fDGF. Renal needle biopsies were performed in 35 out of 177 patients (18%) patients in the UMCG cohort and in 21 out of 200 patients, in the LUMC cohort, within the first 7 days. Within the first year after KTX, a total of 137 (77%) and 129 (65%) of patients received a renal biopsy (including ≤ 14 days biopsies), respectively, in the UMCG cohort and LUMC cohort. In the UMCG cohort, rejection was proven by renal biopsy in 14 out of 177 patients (8%) within the first 7 days and in 57 patients (32%) during the first year (including ≤ 14 days rejections). In the LUMC cohort, rejection was proven in 9 out of 200 (5%) patients within the first 7 days and in 36 patients (18%) during the first year. A significant difference in baseline characteristics was found for the variables ‘age’ (P<0.001), ‘BMI’ (P = 0.025), ‘DGF > 7 days after KTX’ (P<0.001), and biopsy results for 14 days and 1 year after KTX, other baseline characteristics were not significantly different.

**Table 1:** Patient characteristics grouped according to cohort.

	Cohort		
Variable	UMCG cohort (n = 177)	LUMC cohort (n = 200)	P-value
Male ^a^	101 (57)	116 (59)	0.993^c^
Age ^b^	49 ± 14	55 ± 13	<0.001^d^
BMI ^b^	25.6 ± 4.6	26.6 ± 3.7	0.025^d^
Pre-emptive KTX ^a^	17 (10)	24 (12)	0.456^c^
Duration pre-KTX dialysis, months ^e^	32.0 (48.50–54.5)	36.4 (14.3–57.3)	0.425^c^
Type of donation			
Living-(un)related	31 (17)	32 (16)	
DBD	72 (41)	50 (25)	
DCD	74 (42)	94 (47)	
Kidney-Pancreas	0 (0)	24 (12)	
Side of transplant, right ^a^	144 (81)	153 (77)	0.168^c^
Duration of DGF, days ^b^	7 (4–14)	5 (0–9)	0.722^c^
DGF > 7 days after KTX ^a^	143 (81)	131 (66)	<0.001^c^
Biopsies performed ^a^			
7 days after KTX	35 (18)	21 (11)	0.012^c^
14 days after KTX	87 (49)	45 (23)	<0.001^c^
1 year after KTX	137 (77)	129 (65)	<0.001^c^
Rejection ^a^			
7 days after KTX	14 (8)	9 (5)	0.167^c^
14 days after KTX	40 (23)	17 (9)	<0.001^c^
1 year after KTX	57 (32)	36 (18)	<0.001^c^

KTX = kidney transplantation DBD = donation after brain death DCD = donation after circulatory death DGF = delayed graft function.

^a^ n (%).

^b^ mean ± standard deviation(SD).

^c^ P-value by chi-square test.

^d^ P-value by student-t test.

^e^ median (IQR).

### Qualitative grading and DGF prediction

**Fig 3:**
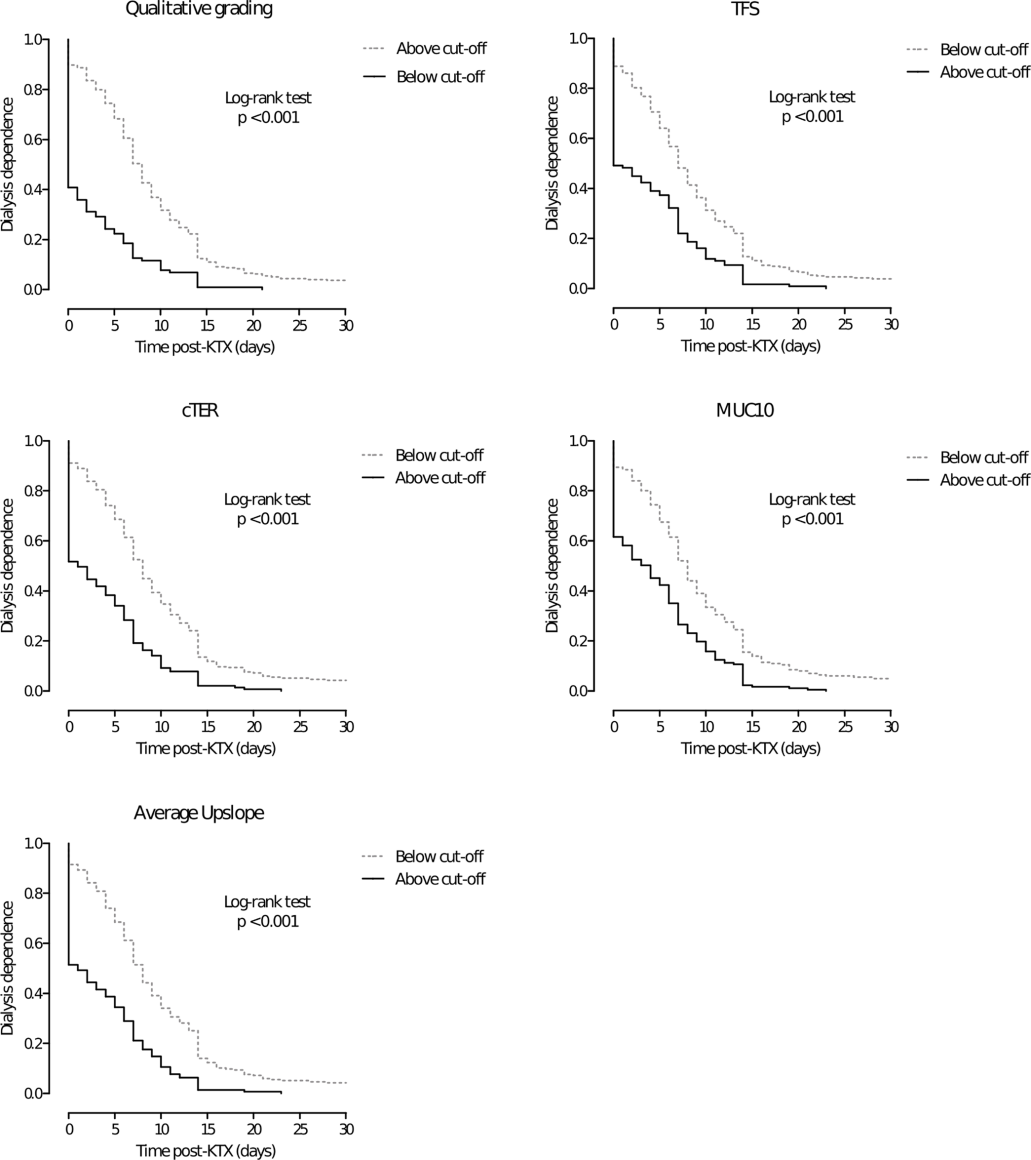
Kaplan-Meier curves, showing the incidence of delayed graft function within the first month after kidney transplantation (censored for patients with DGF > 30 days, n = 10) for (A) qualitative grading, (B) *TFS*, (C) *cTER*, (D) *MUC10*, and (E) *Average upslope*. Higher curves for the group of patients with an expected longer duration of delayed graft function and lower curves for the group of patients with in expected shorter duration of delayed graft function, based on quantitative analyses and qualitative grading.

Results of qualitative grading of the RS curves are shown in Table 2. *Grade 1* pattern was seen in 38 patients (10%), *grade 2* in 65 patients (17%), *grade 3* was seen in 180 patients (48%) and *grade 4* in 94 patients (25%). The duration of DGF increased significantly (P<0.001) with higher Heaf and Iversen grades (Spearman correlation of r = 0.54). The proportion of patients with DGF ≥ 7 increased with higher Heaf and Iversen grades: 10 out of 38 (26%) patients with *Grade 1*, 26 out of 65 (40%) patients with *Grade 2*, 146 (81%) out of 180 patients with *grade 3*, and in 92 (98%) out of 94 patients with *grade 4* pattern experienced DGF ≥ 7. Kaplan-Meier curves (Fig 3), for patients with a qualitative grade above and below the optimal cut-off, were significantly different (log-rank P<0.001). Qualitative grading for the prediction of DGF ≥ 7 days, with > *grade 2* as cut-off, had a sensitivity and specificity of respectively 87% and 65%, with a positive and negative predictive value of 87% and 65%.

**Table 2:** Qualitative and quantitative analysis (sensitivity / specificity / PPV / NPV / ROC) for ≥ 7 days delayed graft function.

Index		Cut-off	Sensitivity, %	Specificity, %	YI	PPV, %	NPV, %	AUC	95% Confidence interval	
									Lower limit	Upper limit
**Qualitative**										
	RS Grade	>2	87	65	0.52	87	65	-	-	-
**Quantitative**										
	*TFS*	0.64	80	61	0.41	84	53	0.75	0.70	0.81
	*cTER*	262	76	72	0.48	88	52	0.80	0.75	0.85
	*MUC10*	825	64	77	0.41	88	44	0.75	0.69	0.80
	*Average upslope*	0.47	75	73	0.48	88	52	0.82	0.78	0.86

RS Grade = Renal scintigraphy grade from 0 to 4; DGF = delayed graft function, YI = Youden-Index, PPV = positive predictive value, NPV = negative predictive value; AUC = area under the curve, TFS = Tubular function slope, cTER = corrected tubular extraction rate, MUC10 = first 10 minutes uptake as a fraction of the injected dose; Average upslope = reflecting the slope during counts at 20 seconds and counts at 3 minutes.

### Quantitative analysis and DGF prediction

Differences in mean values for patients with (n = 274) or without DGF (n = 103) were statistically significant for *cTER*, *Average upslope*, *TFS*, *MUC10* (P<0.001) and *R20/3* (P = 0.042) (Table 3). The duration of DGF increased significantly (P<0.001) with lower quantitative results: *TFS* (r = -0.39), *cTER* (Spearman correlation of r = -.46), *MUC10* (r = -0.39), *Average Upslope* (r = -0.47). *R20/3* results did not significantly correlate with the duration of DGF (P = 0.848). *cTER* and *Average slope* provided a reliable discrimination with AUC values of respectively 0.80 and 0.82 for DGF ≥ 7 (Table 2). The levels of discrimination of *TFS* and *MUC10* were considered acceptable, with an AUC of respectively 0.75 and 0.75 for DGF ≥ 7, and *R20/3* had a poor level of discrimination, with an AUC of 0.57. The optimal cut-off values for parameters with a reliable or acceptable level of discrimination are presented in Table 2. AUC values were not significantly different between patients from the UMCG cohort and LUMC cohort for *TFS* (P = 0.226), *cTER* (P = 0.089), *MUC10* (P = 0.758), and *Average Upslope* (P = 0.096). Log-rank tests comparing Kaplan-Meier curves (Fig 3), for patients with a quantitative grade above and below the optimal cut-off, were significant (P<0.001) for the indices *TFS*, *cTER*, *MUC10*, and *Average Upslope*. YI values of *cTER* and *Average upslope*, for predicting DGF ≥ 7 days, was 0.48 for both indices, with a sensitivity 76% and a specificity of 72% for *cTER*, and a sensitivity of 75% and specificity of 73% for *Average upslope*. The YI for *TFS* and *MUC10*, for predicting DGF ≥ 7 days, was 0.41 for both indices, with a sensitivity 80% and a specificity of 61% for *TFS*, and a sensitivity of 64% and specificity of 77% for *MUC10*. The corresponding positive and negative predictive values for all indices are shown in Table 2.

**Table 3:** Distribution of index values for delayed graft function.

Index	DGF (n = 274)	Non-DGF (n = 103)	P-value
*TFS*	0.30 (0.13–0.53)	0.75 (0.38–1.43)	**<0.001**
*cTER*	144 (70–257)	444 (237–768)	**<0.001**
*MUC10*	644 (393–1069)	1249 (830–2271)	**<0.001**
*Average upslope*	0.23 (0.09–0.48)	0.88 (0.46–1.49)	**<0.001**
*R20/3*	1.68 (1.20–2.09)	1.57 (1.02–1.91)	**0.042**

P-value by Mann-Whitney U test, median (IQR), significant values (two-tailed) are presented in bold, R20/3 = uptake at 20 minutes divided by the uptake at 3 minutes, TFS = Tubular function slope, cTER = corrected tubular extraction rate, MUC10 = first 10 minutes uptake as a fraction of the injected dose, Average upslope = reflecting the slope during counts at 20 seconds and counts at 3 minutes

### Differentiating ATN and rejection

Based on previously published studies, we tried to reproduce whether patients with a DGF course longer than anticipated were more likely to have an additional pathology such as rejection. This group did not show more cases of rejection between day 7 and 14 compared to the group with expected DGF ≥ 7 days, based on qualitative grading: 4 (11%) patients with rejection out of the 36 patients below the cut-off (*grade 2*) and 24 (10%) patients with rejection out of the 238 patients above the cut-off. Excluding all cases of rejection between day 7 and 14, did not result in a better performance for predicting DGF ≥ 7 days, with an AUC of 0.76 (interval = 0.70–0.81) for *TFS*, 0.80 (0.74–0.85) for *cTER*, 0.75 (0.70–0.81) for *MUC10*, 0.82 (0.78–0.87) *Average Upslope*, and a sensitivity and specificity for qualitative grading of 87% and 63%, respectively.

## Discussion

This study shows that a DGF duration of ≥ 7 days post-KTX can be predicted by using qualitative grading of RS curves and RS quantitative indices (*cTER* and *Average upslope*). The RS grades and indices showed a high sensitivity and positive predictive value for predicting a DGF duration longer than 7 days. However, the specificity of DGF prediction is insufficient to reliably identify patients with longer than expected DGF duration and a higher risk of AR. These findings can facilitate clinical management and help to inform patients on the expected course after KTX. RS does not help to identify patients with an increased risk of rejection and requiring renal biopsy.

Several previous studies described the value of qualitative and quantitative RS analyses using Tc-99m MAG3 or Tc-99m DTPA. However, only three of these studies focused on the prediction or correlation of RS curve grading and transplantation outcomes. [14,17,19 In 2000 the qualitative grading scale of Heaf and Iversen for Tc-99m MAG3 RS was introduced first and found a 76% sensitivity for predicting a 10% serum creatinine rise within two days.[14] In two more recent studies, the applicability of a perfusion curve grading scale for Tc-99m DTPA RS was described in which a sensitivity and specificity of respectively 94% and 44% was reported for the prediction of rejection within the first week after KTX and a sensitivity and specificity of 39.5% and 93.7% for the prediction of a serum creatinine rise at three months after KTX. [17,19] Due to differences in Tc-99m DTPA and Tc-99m MAG3 curve shapes, caused by the higher renal extraction rate of MAG3 compared to DTPA, we could not apply this grading scale of Tc-99m DTPA in our study.

Three previous publications focused on DGF in which, among others, a significant correlation between the *MUC10* index and the occurrence of DGF within the first week post-KTX was reported, concluding that a *MUC10* value below the cut-off indicates a poorer graft prognosis. [8,10] In 2002, the *TFS* index was introduced for the use of predicting DGF. In a small prospective study in 42 patients, the authors demonstrated a significant difference in *TFS* values for patients with DGF and patients with immediate graft function.[8] A more recent article by Yazici et al., described a high sensitivity and specificity (86.1% and 86.2%) for the identification of DGF by using the relatively new index *graft index (GI)* in 179 patients.[9] Since our study was focusing on Tc-99m MAG3 RS, we were again unable to apply this new Tc-99m DTPA RS curve index. However, the presence of DGF is generally detected by serum creatinine measurement and RS is only of additional value if it can provide information on the cause of DGF or the prognosis. In the current study, we focused on the prediction of the duration of DGF which showed to have a sensitivity and specificity of the *TFS* for DGF ≥ 7 days of 80% and 61%.

Some limitations of our study need to be addressed. First, the retrospective design of this study creates the risk of selection bias. RS procedures were performed at the physicians’ discretion in the UMCG cohort which contributed to a selection of patients with generally worse early post-transplant outcomes compared to the whole renal transplant population. This was confirmed by the higher number of rejections at day 14 post-KTX together with a higher percentage of patients with DGF after all types of donation, both showing a significant difference compared to the LUMC cohort. The high number of patients with DGF influenced the positive and negative predictive values, which should therefore be judged together with the provided sensitivity and specificity. Second, we had to exclude 55 patients, because the RS-results could not be retrieved, due to incomplete data. However, we do not expect that this has altered our results since the exclusion was due to technical problems and thus not leading to extra selection bias. Third, the long period of inclusion, 2000–2014, contributed to a variety of RS protocols. The main differences were the duration of the scan, between 20 and 40 minutes, and the software used for the RS-analysis. This shortcoming was addressed by shortening the analyzed procedure time, maximum analyzed procedure duration of 20 minutes, and by using the new method for the quantitative analyses. Finally, we do not expect large differences in the injected dose of the radiopharmaceutical since the deviation in administered doses were considered small. Additionally, we minimized the effect of the administered doses by correcting the quantitative indices for the administered dose.

In conclusion, the qualitative RS-grading and the RS quantitative indices *cTER* and *Average upslope* predict a DGF duration longer than 7 days with a high sensitivity. This finding may help to support both clinicians and patients in managing expectations in the early post-operative period. However, due to the low specificity for DGF ≥ 7 days RS does not contribute to the identification of patients with prolonged DGF due to superimposed rejection.

## References

[1] Cornell LD, Smith RN, Colvin RB. Kidney transplantation: mechanisms of rejection and acceptance. Annu Rev Pathol 2008;3:189–220. doi: 10.1146/annurev.pathmechdis.3.121806.151508 18039144 10.1146/annurev.pathmechdis.3.121806.151508

[2] Perico N, Cattaneo D, Sayegh MH, Remuzzi G. Delayed graft function in kidney transplantation. Lancet 2004; 13–19;364(9447):1814–1827. 15541456 10.1016/S0140-6736(04)17406-0

[3] Sharif A, Borrows R. Delayed graft function after kidney transplantation: the clinical perspective. Am J Kidney Dis 201; 62(1):150–158. doi: 10.1053/j.ajkd.2012.11.050 23391536 10.1053/j.ajkd.2012.11.050

[4] Mallon DH, Summers DM, Bradley JA, Pettigrew GJ. Defining delayed graft function after renal transplantation: simplest is best. Transplantation 2013; 96(10):885–889. doi: 10.1097/TP.0b013e3182a19348 24056620 10.1097/TP.0b013e3182a19348

[5] Yarlagadda SG, Coca SG, Formica RN Jr, Poggio ED, Parikh CR. Association between delayed graft function and allograft and patient survival: a systematic review and meta-analysis. Nephrol Dial Transplant 2009; 24(3):1039–1047. doi: 10.1093/ndt/gfn667 19103734 10.1093/ndt/gfn667

[6] Butala NM, Reese PP, Doshi MD, Parikh CR. Is delayed graft function causally associated with long-term outcomes after kidney transplantation? Instrumental variable analysis. Transplantation 2013; 95(8):1008–1014. doi: 10.1097/TP.0b013e3182855544 23591726 10.1097/TP.0b013e3182855544 PMC3629374

[7] Johnston O, O'kelly P, Spencer S, Donohoe J, Walshe JJ, Little DM, et al Reduced graft function (with or without dialysis) vs immediate graft function—a comparison of long-term renal allograft survival. Nephrol Dial Transplant 2006; 21(8):2270–2274. doi: 10.1093/ndt/gfl103 16720598 10.1093/ndt/gfl103

[8] El-Maghraby TA, Boom H, Camps JA, Blokland KA, Zwinderman AH, Paul LC, et al Delayed graft function is characterized by reduced functional mass measured by (99m)Technetium-mercaptoacetyltriglycine renography. Transplantation 2002; 74(2):203–208. 12151732 10.1097/00007890-200207270-00010

[9] Yazici B, Oral A, Gokalp C, Akgun A, Toz H, Hoscoskun C. A New Quantitative Index for Baseline Renal Transplant Scintigraphy With 99mTc-DTPA in Evaluation of Delayed Graft Function and Prediction of 1-Year Graft Function. Clin Nucl Med 2016; 41(3):182–188. doi: 10.1097/RLU.0000000000001020 26447378 10.1097/RLU.0000000000001020

[10] Stevens H, Klerk JM, Mertens IJ, Rijk PP, Hene RJ. Quantitative baseline renography 48 hours after renal transplantation predicts long-term graft survival. Eur J Nucl Med 2001;28(11):1677–1681. doi: 10.1007/s002590100650 11702110 10.1007/s002590100650

[11] Guignard R., Mourad G., Mariano-Goulart D. Utility of postsurgical renal scintigraphy to predict one-year outcome of renal transplants in patients with delayed graft function. Nucl Med Commun 2011;32(4):314–319. doi: 10.1097/MNM.0b013e3283446297 21301375 10.1097/MNM.0b013e3283446297

[12] El-Maghraby T.A.F., De FJ, Wasser M.N.J.M., Pauwels E.K.J. Diagnostic imaging modalities for delayed renal graft function: A review. Nucl Med Commun 1998;19(10):915–936. 10234672 10.1097/00006231-199810000-00002

[13] Russell C.D., Dubovsky E.V., Taylor A.T Jr. Prediction of urinary excretion of technetium-99m-MAG3. J Nucl Med 1998;39(7):1257–1259. 9669405

[14] Heaf JG, Iversen J. Uses and limitations of renal scintigraphy in renal transplantation monitoring. Eur J Nucl Med 2000; 27(7):871–879. 10952501 10.1007/s002590000281

[15] Majima T, Hattori R, Funahashi Y, Komatsu T, Kato M, Yamada S, et al (99m)Tc-mercaptoacetyl triglycine renography to monitor renal transplant function among kidneys from donors after cardiac death. Transplant Proc 2012; 44(1):49–53. doi: 10.1016/j.transproceed.2011.11.028 22310576 10.1016/j.transproceed.2011.11.028

[16] El-Maghraby T.A.F., De FJ, Van Eck-Smit B.L.F., Zwinderman A.H., El-Haddad S.I., Pauwels E.K.J. Renographic indices for evaluation of changes in graft function. Eur J Nucl Med 1998;25(11):1575–1586. 9799356 10.1007/s002590050338

[17] Yazici B., Yazici A., Oral A., Akgun A., Toz H. Comparison of renal transplant scintigraphy with renal resistance index for prediction of early graft dysfunction and evaluation of acute tubular necrosis and acute rejection. Clin Nucl Med 2013;38(12):931–935. doi: 10.1097/RLU.0000000000000271 24152633 10.1097/RLU.0000000000000271

[18] Russell C.D., Yang H., Gaston R.S., Hudson S.L., Diethelm A.G., Dubovsky E.V. Prediction of renal transplant survival from early postoperative radioisotope studies. J Nucl Med 2000;41(8):1332–1336. 10945523

[19] Yazici B, Oral A, Gokalp C, Akgün A, Toz H, Ozbek SS, et al Evaluation of renal transplant scintigraphy and resistance index performed within 2 days after transplantation in predicting long-term graft function. Clin Nucl Med 2015; 40(7):548–552. doi: 10.1097/RLU.0000000000000789 25899587 10.1097/RLU.0000000000000789

[20] Li Y, Russell CD, Palmer-Lawrence J, Dubovsky EV. Quantitation of renal parenchymal retention of technetium-99m-MAG3 in renal transplants. J Nucl Med 1994; 35(5):846–850. 8176469

[21] Hosmer DW, Lemeshow S, Sturdivant RX. Applied Logistic Regression. 3th ed Chicester: Wiley; 2013.

[22] Shan G. Improved Confidence Intervals for the Youden Index. PLoS One 2015 7 1;10(7):e0127272 doi: 10.1371/journal.pone.0127272 26132806 10.1371/journal.pone.0127272 PMC4488538

